# Anthropomorphic Strategies Promote Wildlife Conservation through Empathy: The Moderation Role of the Public Epidemic Situation

**DOI:** 10.3390/ijerph18073565

**Published:** 2021-03-30

**Authors:** Dan Yue, Zepeng Tong, Jianchi Tian, Yang Li, Linxiu Zhang, Yan Sun

**Affiliations:** 1 Key Laboratory of Behavioral Science, Institute of Psychology, Chinese Academy of Sciences, Beijing 100101, China; leewui_cn@163.com (D.Y.); tongzp@psych.ac.cn (Z.T.); tjc0327@163.com (J.T.); 2 Department of Psychology, University of Chinese Academy of Sciences, Beijing 100049, China; 3 Business School, Beijing Technology and Business University, Beijing 100048, China; girlhelen616@126.com; 4 Laboratory of Ecosystem Network Observation and Simulation, Institute of Geosciences and Resources, Chinese Academy of Sciences, Beijing 100101, China; lxzhang.ccap@igsnrr.ac.cn; 5 The United Nations Environment Programme—International Ecosystem Management Partnership, Beijing 100101, China

**Keywords:** public epidemic, negative emotion, anthropomorphism, wildlife conservation, empathy

## Abstract

The global illegal wildlife trade directly threatens biodiversity and leads to disease outbreaks and epidemics. In order to avoid the loss of endangered species and ensure public health security, it is necessary to intervene in illegal wildlife trade and promote public awareness of the need for wildlife conservation. Anthropomorphism is a basic and common psychological process in humans that plays a crucial role in determining how a person interacts with other non-human agents. Previous research indicates that anthropomorphizing nature entities through metaphors could increase individual behavioral intention of wildlife conservation. However, relatively little is known about the mechanism by which anthropomorphism influences behavioral intention and whether social context affects the effect of anthropomorphism. This research investigated the impact of negative emotions associated with a pandemic situation on the effectiveness of anthropomorphic strategies for wildlife conservation across two experimental studies. Experiment 1 recruited 245 college students online and asked them to read a combination of texts and pictures as anthropomorphic materials. The results indicated that anthropomorphic materials could increase participants’ empathy and decrease their wildlife product consumption intention. Experiment 2 recruited 140 college students online and they were required to read the same materials as experiment 1 after watching a video related to epidemics. The results showed that the effect of wildlife anthropomorphization vanished if participants’ negative emotion was aroused by the video. The present research provides experimental evidence that anthropomorphic strategies would be useful for boosting public support for wildlife conservation. However, policymakers and conservation organizations must be careful about the negative effects of the pandemic context, as the negative emotions produced by it seems to weaken the effectiveness of anthropomorphic strategies.

## 1. Introduction

As globalization continues to grow, the business model of transnational trade is becoming increasingly mature, which led to the rapid growth of legal and illegal wildlife trade worldwide [1,2]. Meanwhile, it was pointed out that the value of total wildlife trade in China’s imports under the control of the Convention on International Trade in Endangered Species of Wild Fauna and Flora rose from 260 million to 1.45 billion [3].

Wildlife resources are the material basis for human survival and development [4]. However, driven by the lure of huge profits, the illegal wildlife trade has become the third largest crime in the world after weapons and drugs, which should be severely punished [5]. It not only directly threatens biodiversity and the survival of endangered species [6], but also leads to the appearance of invasive alien species [7] and infectious disease transmission [8]. According to American biogeographer Jared Diamond [9], a pandemic originating in wildlife is a “fatal gift from animal friends.” In other words, wild animals who lost their ecological barriers became mobile virus banks [10]. As we could see, COVID-19 was identified as having originated in wildlife sources [11]. Several epidemiological studies speculated that bats or pangolins could be the intermediate hosts during virus transmission and mutation [12,13,14,15]. In fact, most new infectious diseases in the past ten years were zoonotic diseases, such as SARS and MERS. According to academic statistics, 60.3% of 335 new infectious diseases broke out in the world between 1940 and 2004, and 71.8% of them originated from wild animals [16]. The negative ecological and social impacts of wildlife trade and wildlife product consumption are magnified in today’s global environment. However, addressing illegal wildlife trade and associated security risks requires a fundamental reduction in market demand, especially for wildlife meat consumption and other non-essential living needs. Therefore, it is necessary to understand the factors influencing such needs to provide academic support for changing wildlife consumption behavior. In recent years, scholars and NGOs called for intervention in the behavior of consumers to curb the demand for wildlife and thereby fundamentally weaken the thriving illegal wildlife trade [17,18]. Currently, consumer-oriented interventions usually include measures like judicial regulations, environmental education, and public awareness, but their effectiveness is barely satisfactory. For example, the revision and innovation of judicial regulations is a very slow process. Thus far, the Law of the People’s Republic of China on the Protection of Wild Animals does not specify the inspection and quarantine rules for many edible wild animals. Additionally, even the SARS outbreak did not do much to build up public awareness that people should reduce the consumption of wild meat. In a word, it is an urgent need to adopt some new approaches and employ a combination of intervention strategies to change the wildlife consumption behavior of citizens.

From the view of public welfare promotion and science education, in recent years, many scholars agreed that the use of anthropomorphism can effectively improve people’s pro-social behavior tendency [19]. Many empirical studies showed that anthropomorphism had a positive effect on promoting individual green consumption behavior [20] and environmental awareness [21]. However, only a few studies discussed attitude toward wildlife [22,23], and the underlying psychological mechanism that can explain the effect of anthropomorphism on individual behavior is still unknown. Reducing outbreak and spread of zoonotic diseases has become an important public health issue worldwide, and curbing wildlife consumption and increasing public willingness to protect wildlife is the key to solving this problem. Therefore, in the context of the global pandemic, this study analyzes the intervention effect of anthropomorphism on wildlife consumption behavior through empirical research, and attempts to explore the psychological mechanism that drives this effect.

## 2. Theoretical Background

### 2.1. Anthropomorphizing

At least as early as the late 6th to early 5th century BC, the Greek philosopher-poet Xenophanes had discussed anthropomorphism. In the last few centuries, anthropomorphism attracted the attention of psychologists, sociologists, and philosophers alike. In particular, social psychological research proposed numerous insights into the nature and consequences of anthropomorphism. In 2007, Epley [24] defined anthropomorphism as the tendency to connect human characteristics, intentions, motivations, or emotions with nonhuman agents. These nonhuman agents include natural forces, electronic devices, religious deities, and non-human animals [25].

With further research, scholars from many fields determined that anthropomorphism is a natural tendency of human beings [26,27,28]. As the Oxford Dictionary puts it [29], “anthropomorphism is the attribution of human characteristics or behavior to a god, animal, or object.” The actions and thoughts of humans can be fluently understood by people because they are the most familiar concepts that people know [30]. For this reason, people tend to interpret non-human agents as humanlike throughout their lives [30]. In addition, a series of studies proposed that anthropomorphizing non-human entities universally occurred in both adults and children [31,32,33] Epley et al. [24] showed that anthropomorphism is most likely to occur in the following three situations: (1) Anthropocentric knowledge is activated by the correlative mind and situation. (2) The interaction between people and non-human agents is effectively motivated. (3) Individuals lack contact with other humans for a long time. Several studies support these theories [31,34,35,36,37]. More generally, these three reasons point to anthropomorphism as a process with functional value [30]. Thus, anthropomorphism might confer some benefits from a psychological perspective.

In fact, Hume [27] found that anthropomorphism can be considered a universal phenomenon in which people anthropomorphize nonhuman agents even unconsciously. Anthropomorphism focuses not only on the appearance characteristics of humans, but also on the human mind. Furthermore, research shows that people naturally anthropomorphize inanimate objects in order to form social connections or control unpredictable objects [35,36]. Guthrie [38] believed that anthropomorphism is a cognitive strategy for understanding the world. For instance, the phenomenon of anthropomorphism of the natural environment is everywhere in daily life. For example, the concept of “Mother Earth” is widely used by various environmental organizations in publicity campaigns. In existing research, most practical applications of anthropomorphism focus on the fields of brand and product promotion [39] and human–computer interaction design [40]. In marketing for children, previous research showed that anthropomorphized depictions effectively promoted the relationship between the child and the product [41]. The purpose of these studies is to answer questions about how to improve brand reputation, how to increase product sales, and how to humanize product design.

### 2.2. Anthropomorphizing in Wild Animal Conservation

In the last few years, the effect of anthropomorphizing environmental objects attracted the attention of many researchers [19,20]. Existing studies report that we tend to be reluctant to hurt a non-human being or object when we think of it as having senses and feelings [25]. Similarly, when people believe that nature has emotions, they are more likely to care about it [42]. Therefore, the “Mother Earth” phrase might be a universal example of anthropomorphizing nature. In other words, an anthropomorphized nature might be more likely to evoke sympathy [19].

Anthropomorphization of animals is generally understood as the cognitive process of assigning human characteristics to animals, such as mind, secondary emotions, and high-level analytical capabilities [43]. Previous study showed that anthropomorphism not only affects empathy [44,45], but can also boost public support for wildlife conservation [20]. Generally, the more people believe that nonhuman agents have senses and feelings, the less they want to hurt them [25]. For example, people will be more reluctant to eat anthropomorphic animals [46], and even give more help to dogs whose close relatives are anthropomorphized [47]. It follows that anthropomorphism is a valuable conservation tool for animals [48]. Anthropomorphism is of practical significance in most social fields because it turns nonhuman agents into moral agents who are worthy of respect. Anthropomorphized representations of animals cause people to pay increasing attention to animal welfare [47]. It also helps us to do our utmost to protect our rich biodiversity and ecosystems, which is the significance of our study of anthropomorphism in the field of wildlife consumption and protection [49].

### 2.3. The Negative Emotions Aroused by the Public Epidemic

COVID-19 lasted for more than a year and is still spreading around the world. Due to the tragic consequence of a public epidemic, people have a negative emotional response to the epidemic. The motivational orientations are activated by emotional or affective states, which means that approach and avoidance behavior is influenced by emotion, and the startle response behaviors are increased when negative emotion is elicited in humans and animals [50]. Approach behavior is a preparation to increase the connection between the individual and objects that include the physical and mental action. On the contrary, the consequence of avoidance behavior is moving away from a target in the environment. However, the main function of anthropomorphic strategies is to decrease the distance of individual and non-human objects. Therefore, from the perspective of motivation, anthropomorphic strategies will conflict with the public epidemic context. An individual who is affected by anthropomorphic strategies and receives information about public epidemic contents might no longer have the intention of wildlife conservation. In addition, previous research indicates that the negative information gets more attention due to the “negative bias” effect in humans and animals [51]. For example, anti-drunk-driving information commonly strikes a negative tone to arouse fears among viewers. Given the above, the present research assumes that the effectiveness of anthropomorphic strategies for wildlife conservation is weakened by the public epidemic context.

## 3. Experiment 1

Experiment 1 has two-fold purposes. First, we aim to explore whether anthropomorphic materials could reduce the consumption intention of wildlife products and generate more wildlife conservation intentions than non-anthropomorphic wildlife content. Second, we attempted to discover the underlying psychological process which is the driving force behind anthropomorphism.

### 3.1. Experimental Materials

#### 3.1.1. Anthropomorphic Wildlife Photographs

All photographs of Experiment 1 presented common wildlife images based on the literature and real advertisement situations to ensure that the manipulation was successful (e.g., tiger, elephant, etc.). The present study invited some experts and postgraduate students in the field of psychology to discuss the selection of photographs. After the discussion of the research group, 30 photographs of wildlife were shortlisted.

The differences in materials between the anthropomorphic and control conditions were the actions and postures of wildlife. The wildlife in the pictures of anthropomorphic materials was similar to human beings in appearance, posture, and movement, but the wildlife in non-anthropomorphic materials were in their normal state. To verify the experimental materials, 33 participants (18 males) were recruited for a pilot study. All participants in the pilot study were asked to look at the 30 wildlife photographs and rate each picture. The question was, “How similar do you think the wildlife in the photograph are to human beings?” This item was rated on a 7-point scale (1 = “very dissimilar” and 7 = “very similar”). A total of eight photographs were selected as the final experimental materials, according to the mean value of the original photographs; the four photographs with the highest score and the other four photographs with the lowest score were assigned to the two conditions. Table 1 shows the scores of these eight pictures and the details of this pilot study.

#### 3.1.2. Text Content in Anthropomorphic Wildlife Materials

Combining pictures with texts was a common practice in previous studies [20]. Thus, anthropomorphism text materials were added to the experiment. We referred to previous research for the content of the photographs, and the research group discussed and sorted out the following texts to be included in the anthropomorphic wildlife materials. In contrast, we removed the names, relationships, and even the emotional feelings of wildlife in the texts of the control condition. The text below is the content shown to the experimental group.

*Please read the following story: The main character in the story is the tiger cub in the picture above. After reading, please fill in the questionnaire and express how you feel.*

*‘Little tiger cub Bobby and his mother live in a cave. At the foot of the cave lives Bobby’s friend, elephant Mars. One day, Bobby’s mother went out and found some food for him. However, Bobby has been waiting for a long time in the cave and even wanted to go out and look for his mother. Bobby was very hungry, and he sought his mom with elephant Mars. However, Bobby and Mars were unfortunately found by poachers in the forest, and the poachers caught them. When Bobby was brought back to the camp by poachers, he saw a tiger skin with a notch in the ear, and he realized that his mom was dead. Just then, the poacher aimed his rifle at Bobby….’*

#### 3.1.3. Empathy

The present study examined empathy as a mediator to explain the effect of anthropomorphism. Empathy was measured using the scale from Batson et al [52], and six items were finally used in the present study. The items were as follows: “I feel sympathetic/warm/compassionate/softhearted/tender/moved for the little tiger cub” from 1 = “Not at all” to 7 = “Extremely” (Cronbach α = 0.93).

#### 3.1.4. Wildlife Product Consumption Intention

The study measured the wildlife product consumption intention using a six-item scale from a previous study [53]. The items were: ‘What is your attitude towards other people’s purchase of wildlife products?’/‘To what extent are you willing to recommend relatives or friends to purchase wildlife products?’/‘What is your attitude toward other people’s purchase of ivory bracelets?’/‘To what extent are you willing to recommend relatives or friends to purchase wildlife products?’/‘What is your attitude toward other people’s purchase of tiger bone wine?’/‘To what extent are you willing to recommend relatives or friends to purchase tiger bone wine?’ with ‘1′ = “Strongly disagree” to ‘7′ = “Strongly agree” (Cronbach α = 0.84).

#### 3.1.5. Wildlife Conservation Intention

The scale measuring wildlife conservation intention was adopted from Herzog et al. [54], and three items were finally determined: ‘*To what extent are you willing to learn about wildlife conservation?’/‘To what extent are you willing to spread wildlife conservation knowledge to others?’/‘To what extent are you willing to donate money if a wildlife conservation foundation is raising money to protect wildlife.*’ These items ranged from ‘1′ = “strongly reluctant” to ‘7′ = “strongly willing” (Cronbach α = 0.84).

### 3.2. Participants

A total of 262 college students were recruited from an online survey platform in Beijing, and 17 participants were removed for failing to complete the questionnaire on time (too quickly or too late). Thus, a total of 245 participants (66.5% female; 87.3% aged 20–29 years) completed the experiment, and a gift was given to these participants. There were two conditions in Experiment 1 (anthropomorphism vs. non-anthropomorphism), and all participants were randomly assigned to one of the two conditions.

### 3.3. Procedure

All participants were informed of the purpose of the experiment, and they were required to read the relevant materials. Then, participants were required to rate their wildlife product consumption intention, wildlife conservation intention, and empathy, on each scale. In addition, all participants were asked to provide demographic information about gender, age, etc.

### 3.4. Results

The results indicated that the main effect of anthropomorphism was not significant (*t* = 1.14, *p* > 0.05). In addition, the difference of wildlife products consumption intention and wildlife protect intention between the anthropomorphic condition and control condition was not significant. However, there was a significant difference (*t* = −4.16, *p* < 0.01) in empathy between the anthropomorphic condition (M = 6.04, SD = 1.04), and control condition (M = 5.36, SD = 1.46).

The correlation analysis results showed that there was a significant positive correlation between anthropomorphism and empathy (r = 0.26, *p* < 0.01). Further, empathy was negatively correlated with the intention to consume wildlife products (r = −0.26, *p* < 0.01) and positively correlated with wildlife conservation intentions (r = 0.47, *p* < 0.01). The correlation results suggest that anthropomorphism could have affected the wildlife product consumption intention and wildlife conservation intention through the individual empathy state. Table 2 shows the correlation analysis results for all variables.

Additionally, the results indicated that the gender difference for wildlife consumption intention (Mmale = 1.63, SD = 0.77; Mfemale = 1.56, SD = 0.75; *p* > 0.05) and protection intention (Mmale = 5.74, SD = 1.09; Mfemale = 5.58, SD = 1.00, *p* > 0.05) was not significant. And there was no difference in ages.

Additionally, the present study used the PROCESS method of Hayes (2013), taking wildlife product consumption intention and wildlife conservation intention as the dependent variables, and Model 4 was selected to conduct 5000 bootstrap samples at the 95% confidence interval. The mediating effect of empathy on wildlife product consumption intention was significant (LLCL = −0.139, ULCI = −0.013, excluding zero). In addition, the mediating effect of empathy on wildlife conservation intention was significant (LLCL = 0.092, ULCI = 0.332, excluding zero). The results of the analysis for the mediating effect showed that the empathy state mediated the influence of anthropomorphic wildlife photographs and texts on wildlife product consumption intention and wildlife conservation intention. Table 3 and Table 4 present the results.

### 3.5. Discussion

According to the results of Experiment 1, we suggest that anthropomorphic wildlife photographs and texts could significantly influence individual empathy states. Further, empathy could negatively predict the individual wildlife consumption intention, and positively predicted wildlife conservation intention. The results of Experiment 1 suggested that anthropomorphism highlighted the similarity between wildlife and humans, which could increase the empathy level of the consumer, and affect their attitude accordingly. One might argue that the context that influences consumers’ emotions might have a bearing on the effect of anthropomorphism. It is important because we need to define the boundary of our proposed mechanism. Thus, experiment 2 was designed to explore how the current pandemic influences the effects of anthropomorphism.

## 4. Experiment 2

The purpose of Experiment 2 was to test the interactive effect of negative emotional states and anthropomorphism. Additionally, Experiment 2 provided further support to the underlying role of empathy.

### 4.1. Experimental Materials

#### 4.1.1. Negative Emotional Video Materials

Considering that Experiment 2 features the pandemic as the social background, it was anticipated that it would probably be difficult to elicit a high degree of involvement from the participants. In order to improve the involvement of participants in the experiment and increase the authenticity of experimental materials, the present study used video materials, which was proven to be an effective approach to induce emotions as a negative emotional cue. Two videos were selected as negative and neutral emotional material after discussion with the research group. To test the effectiveness of the videos, we conducted a pilot experiment before our formal experiment. Twenty-five participants (12 males) were recruited for this pilot experiment and all were asked to watch one video on the first day and another video on the second day, with the order being randomized. All participants should complete the questionnaires of negative emotion before and after watching the video. In addition, the present study adapted the measure of negative emotion from the Positive Affect and Negative Affect Scale [55] with 10 items (“After watching the video, to what extent did you feel fearful/scared/sad/anxiety and etc.”), and the scoring system was ‘1′ = “Not at all” to ‘5′ = “Extremely” (Cronbach α = 0.82). The results showed that the negative emotion of participants after watching the negative emotional video was significantly stronger than before (M _After watching_ = 28.92 ± 6.84, M _Before watching_ = 24.28 ± 5.87, *t* = −2.94, *p* < 0.01), but there was no significant difference after watching the neutral emotional material. Table 5 presents the details.

#### 4.1.2. Anthropomorphic Wildlife Photographs and Texts

To ensure the consistency of the experiment, the experimental materials of anthropomorphic wildlife photographs and texts were the same as those used in Experiment 1.

#### 4.1.3. Measures

The measures of empathy and wildlife conservation intention were the same as that in Experiment 1. Experiment 2 was carried out during the COVID-19 period, when the Chinese government introduced laws to strictly control wildlife product consumption and the media also had a lot of publicity about the abuses of wildlife product consumption. Thus, there was a possibility of consumers not showing their true attitude toward wildlife products, due to their awareness that wildlife consumption is not approved by the government and society, further, the distribution of their scores on wildlife products consumption intention might be skewed (e.g., generally, their scores are lower). In sum, the present study did not measure the intention to consume wildlife products in Experiment 2.

#### 4.1.4. Participants

A total of 158 college students were recruited from an online survey platform in China, in response to the promise of a gift in exchange of participation. Eighteen participants did not complete the questionnaire or completed the experiment too quickly, thus, leaving a total of 140 valid participants in this experiment (58.7% female; 86.4% aged 20–29 years)

### 4.2. Procedure

Experiment 2 used a 2 (anthropomorphism vs. non-anthropomorphism) × 2 (negative emotional state vs. neutral emotion state) between-subjects design to examine the effect of negative emotion. Participants were informed that they were completing a study on environmental conservation, and all of them were shown the experimental stimulus under the corresponding conditions. Participants were asked to watch the negative [neutral] emotion video, wildlife photographs, and texts. After this, they completed the questionnaire. The other procedure was similar to that in Experiment 1.

### 4.3. Results

The results of the two-way ANOVA revealed that the main effect of anthropomorphism (F = 18.26, *p* < 0.01, η^2^ = 0.12) and the negative and neutral emotion states (F = 15.30, *p* < 0.01, η^2^ = 0.10) were statistically significant. Additionally, the interaction effect of anthropomorphism and negative and neutral emotion states was significant (F = 6.21, *p* < 0.01, η^2^ = 0.04). As expected, the results of the simple effect analysis showed that the empathy state of participants who read anthropomorphic wildlife photographs and text was significantly higher than participants who read non-anthropomorphic wildlife materials (ΔM = 1.05, *p* < 0.01), but no significant difference was observed between the anthropomorphic and control conditions, following the viewing of the negative and neutral videos (ΔM = 0.28, *p* = 0.20). Table 6 presents the details.

**Figure 1:**
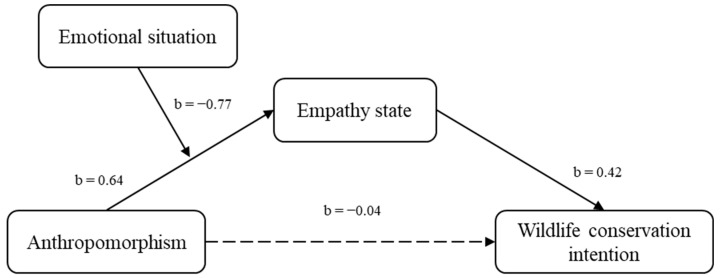
The interaction effect of emotional state on anthropomorphism and wildlife conservation intention (Notes: The dotted line indicates that the path coefficient is not significant).

As expected, anthropomorphism does not have an impact on the empathy state of participants in a negative emotional state. Hence, the emotional state moderates the relationship between anthropomorphism and empathy. We used PROCESS from Hayes [56] to test the moderating role of negative/neutral emotion states, and Model 7 was selected. Anthropomorphism was the independent variable, wildlife conservation intention was the dependent variable, empathy state was the mediating variable, and negative/neutral emotional state was the moderating variable. The results revealed that anthropomorphism had a significant positive effect on empathy (b = 0.64, *t* = 4.71, *p* < 0.01), and empathy had a significant positive effect on wildlife conservation intentions (b = 0.42, *t* = 6.12, *p* < 0.01). The conditional indirect effect revealed that for the participants in the negative emotional state, the indirect effect of empathy state on wildlife conservation intention was not significant (LLCL = −0.04, ULCI = 0.283, including zero). However, for the participants in the neutral emotional state, the indirect effect of empathy on wildlife conservation intention was positive and significant (b = 0.44, SE = 0.13, LLCL = 0.214, ULCI = 0.706 (excluding zero)). Further, the index of moderated mediation was significant (index = −0.32, SE = 0.14, LLCL = −0.62, ULCI = −0.066 excluding zero). Finally, the direct effect of anthropomorphism on wildlife conservation intention was not significant (LLCL= −0.317, ULCI = 0.232, including zero). In other words, the results supported the hypothesis of the present study, in that the interaction effect of negative/neutral emotional state on anthropomorphism and wildlife conservation intention was fully mediated by empathy. Figure 1 shows the results model of Experiment 2.

### 4.4. Discussion

Experiment 2 provides further evidence to the mediating role of empathy in the relationship between anthropomorphism and wildlife conservation intention. In addition, Experiment 2 also revealed that the mediating effect of empathy was more salient for the neutral condition than the participants in the negative emotion conditions, namely the effect of anthropomorphism was impacted by the emotional state.

## 5. General Discussion

Through two experimental studies, we primarily examined the positive influence of anthropomorphism on wildlife conservation. Additionally, we explored the underlying psychological process that drives the effect. More importantly, we discovered how the pandemic weighs in on the effect of anthropomorphized wildlife conservation methods. We found that the effect would no longer exist if participants were immersed in negative emotions induced by the pandemic. Our research contributed to the current literature in terms of the following points.

First, the results of Experiment 1 revealed that anthropomorphism highlights the similarity between wildlife and human. To some extent, giving animals more psychological abilities through anthropomorphization could provide the basis for enhancing the emotional connection between participants and wildlife agents. It is worth noting that empathy state plays a role in the effect of anthropomorphism on wildlife conservation. Previous studies [57,58,59] focused on specific factors such as empathy and belief about animals’ psychological experiences, which are directly related to attitudes toward animals. The more people believe that nonhuman agents have senses and feelings, the less they want to hurt them [25]. For instance, some scholars suggest that people are more reluctant to eat anthropomorphic animals [46]. In addition, previous research pointed out that people are more concerned about animals when considering the emotions of animals [60,61]. Therefore, the results of current study provided another proof that anthropomorphic strategies are useful for wildlife conservation by inducing individuals’ empathy states. Moreover, given the effect of anthropomorphism might be influenced by different emotional states, Experiment 2 further examined the interactive effect of the negative emotional state and anthropomorphism. The results of Experiment 2 showed that the interaction between anthropomorphism and emotion has a significant effect on empathy and wildlife conservation intention. As expected, negative emotion weakened the effect of anthropomorphism on both empathy and intention. Based on previous studies, we noticed that negative emotion might show an ineffective effect in intervention. Our findings further verified that emotion as a boundary condition affect the anthropomorphic intervention.

Last but not the least, current study adopted a sample of students to focus more on the mechanism of the effect of anthropomorphism with emotions rather than other factors (e.g., age, culture). Considering that previous studies showed that individuals’ characteristics and social context are potential factors, we attempted to control these variables as possible. For example, Conrad et al [62] evaluated the impact of anthropomorphic storybooks and animals’ experiences on children, and combined with an adult comparison group. They found the consistency between children and adults, but to some extent, adults showed the effect of anthropomorphism in more aspects. In our study, participants were limited to adults to avoid the underlying influence of age. Meanwhile, our findings also provide a new evidence that anthropomorphism can induce adults’ empathy and boost adults support for wildlife conservation.

## 6. Policy Implications

At present, global illegal wildlife trade has become a critical issue that can no longer be ignored. Especially, after the outbreak of COVID-19 in 2020, in order to avoid the loss of endangered species and safeguard public health, it is worthwhile to work on ending illegal wildlife trade and educating the public on the importance of wildlife conservation.

Interestingly, previous studies showed that anthropomorphism seems to be a new option to encourage people to support wildlife conservation. Government agencies perhaps could integrate anthropomorphism into strategies, when it comes to intervention in wildlife conservation or other fields. Current evidence suggests that anthropomorphism does influence individuals’ wildlife products consumption intention, but the effect of anthropomorphism is not suitable for all situations. For example, if information on the pandemic situation (fear appeal, negative emotion) is included in the application, the effect of anthropomorphic advertisement is greatly reduced. We suggest that policymakers should carefully consider the occasion when they attempt to make use of anthropomorphism to induce individual’s empathy on wildlife. Additionally, as we can see, the effect of anthropomorphism on wildlife conservation only plays a role when people are in neutral or even positive emotional state rather than a negative emotional state. Looking at it from another angle, at least, during the COVID-19 period, it is not advisable to publicize wildlife conservation using anthropomorphism. When we are unable to control the external context, we believe that policymakers can consider how to influence individuals’ empathy states. On the other hand, government agencies also need to pay attention to cultivating empathy through family education, which is a key way to improve public awareness toward wildlife conservation.

All in all, anthropomorphism is a powerful means of evoking and altering individual empathy levels, but it might not be always appropriate across all situations. In the COVID-19 context, for example, it might be a better choice to consider other ways to change the individual empathy state.

## 7. Open Questions and Future Directions

In the last few decades, anthropomorphism attracted the attention of sociologists, philosophers, and psychologists alike. However, most research concentrated on three domains of anthropomorphism—business, human–computer interaction, and law. Studies exploring the psychological mechanism behind it and how it affects individual behavior are limited.

This study expands on empirical demonstrations of anthropomorphizing wild animals as a means of increasing public support for wildlife conservation. It proved the effectiveness of anthropomorphism in altering wildlife consumption behavior intention, and further clarified its psychological mechanism.

The COVID-19 outbreak brought the need for wildlife conservation to the center of public attention. This study showed that anthropomorphism can reduce individual consumption intention and increase individual protection intention by influencing individual empathy states. However, when combined with stimuli on pandemic information, anthropomorphization of wildlife does not produce any significant advantages in changing people’s attitudes toward consumption or protection.

In summary, wildlife anthropomorphization is a very interesting and relatively unexplored topic. Future research can also explore the impact of other factors (such as age, occupation, ethnicity, culture, or other methods of behavior change) on the effect of anthropomorphization on wildlife conservation intention, by integrating more perspectives and adopting more diverse research methods.

The COVID-19 pandemic reminded us that as human beings we need to reflect on our relationship with other beings on this planet, restore the traditional reverence for nature, and maintain the ecological balance between man and nature, because wildlife conservation will ultimately benefit human beings and increase public health security.

## Figures and Tables

**Table 1:** The results for anthropomorphic photograph verifications.

Conditions	Photographs	M	SD
Anthropomorphic condition	1	5.33	2.6
	2	5.27	2.52
	3	5.09	2.65
	4	5.00	2.69
Control condition	5	2.48	2.13
	6	2.42	2.25
	7	2.39	1.75
	8	2.33	1.60

(Notes: *N* = 33).

**Table 2:** Correlation matrix of Experiment 1.

	1	2	3	4
AN	1			
WPCI	0.07	1		
WCI	0.03	−0.29 **	1	
ES	0.26 **	−0.26 **	0.47 **	1

Note. *N* = 245. ** *p* <0.01. AN—anthropomorphism; WPCI—wildlife product consumption intention; WCI—wildlife conservation intention; and ES—empathy state.

**Table 3:** The mediating effect of anthropomorphism on wildlife product consumption intention.

	Model 1 (ES)		Model 2 (WPCI)	
Predictors	b	T	b	*t*
AN	0.678	4.204 ***	0.114	1.141
ES			−0.097	−2.521 *
R^2^	0.068		0.027	
F	17.675 ***		3.303 *	

Note. *N* = 245. * *p* < 0.05. *** *p* < 0.001. AN—Anthropomorphism; ES—Empathy state; and WPCI—Wildlife product consumption intention.

**Table 4:** The mediating effect of anthropomorphism on wildlife conservation intention.

	Model 1 (ES)		Model 2 (WCI)	
Predictors	b	T	b	*t*
AN	0.678	4.204 ***	−0.141	−1.103
ES			0.292	5.948 ***
*R^2^*	0.068		0.128	
F	17.675 ***		17.793 *	

Note. *N* = 245. * *p* < 0.05. *** *p* < 0.001. AN—anthropomorphism; ES—empathy state; and WCI—wildlife conservation intention.

**Table 5:** The results of the pilot study for video materials.

	Negative Emotion		
Conditions	Before	After	*t*
Negative emotional video	24.28 ± 5.87	28.92 ± 6.84	−2.94 **
Neutral emotional video	24.96 ± 6.39	27.64 ± 8.14	−1.51

(Notes: *N* = 25, ** *p* < 0.01).

**Table 6:** The interaction effect of anthropomorphism and negative and neutral emotion states on empathy.

Conditions		Empathy State	
		M	SD
Anthropomorphic condition	Negative emotional state	6.10	0.74
	Neutral emotional state	5.88	0.78
Control condition	Negative emotional state	5.82	0.89
	Neutral emotional state	4.83	1.21

## Data Availability

All data were uploaded on the Figshare. Other researchers can download the dataset in https://figshare.com/s/b863e4410afa0cf06475.
